# Cytotoxic T-cell precursor frequencies to HER-2 (369 – 377) in patients with HER-2/neu-positive epithelial tumours

**DOI:** 10.1038/sj.bjc.6601244

**Published:** 2003-09-09

**Authors:** P A Sotiropoulou, S A Perez, E G Iliopoulou, I Missitzis, V Voelter, H Echner, C N Baxevanis, M Papamichail

**Affiliations:** 1 Cancer Immunology and Immunotherapy Center, Saint Savas Cancer Hospital, 171 Alexandras Avenue, 115 22 Athens, Greece; 2 Breast Cancer Clinic, Saint Savas Cancer Hospital, 171 Alexandras Avenue, 115 22 Athens, Greece; 3 Abteilung Fuer Physicalische Biochemie des Physiologisch-Chemisches Institut der Universitat, 4 Hoppe-Seyler Strasse, 72076 Tuebingen, Germany

**Keywords:** HLA-A3, HLA-A26, cancer patients, prostate cancer, T-cell precursor frequencies, HER-2/neu peptides

## Abstract

HER-2/neu oncoprotein contains several major histocompatibility complex class I-restricted epitopes, which are recognised by cytotoxic T lymphocyte (CTL) on autologous tumours and therefore can be used in immune-based cancer therapies. Of these, the most extensively studied is HER-2(9_369_). In the present report, we used dendritic cells pulsed with HER-2(9_369_) to stimulate, in the presence of IL-7 and IL-12, the production of IFN-*γ* by patients' CTL detected by the enzyme-linked immunosorbent spot-assay. Frequencies of peptide-specific precursors were estimated in HLA-A2, HLA-A3 and HLA-A26 patients with HER-2/neu-positive (+) breast, ovarian, lung, colorectal and prostate cancers and healthy individuals. We found increased percentages of such precursors in HLA-A2 (25%) and HLA-A26 (30%) patients, which were significantly higher (60%) in HLA-A3 patients. Our results demonstrate for the first time that pre-existing immunity to HER-2(9_369_) occurs in patients with colorectal, lung and prostate cancer. They also suggest that HER-2(9_369_) can be recognised by CTL, besides HLA-A2, also in the context of HLA-A3 and HLA-A26, thus increasing the applicability of HER-2(9_369_)-based vaccinations in a considerably broader patients' population.

The HER-2/neu protein is a member of the tyrosine kinase family of growth factor receptors (Coussens *et al*, 1985; Bargmann *et al*, 1986). It is frequently amplified and overexpressed in breast (Slamon *et al*, 2001), ovarian (Slamon *et al*, 1987), pancreatic (Yamanaka *et al*, 1993) and colorectal (Maxwell-Armstrong *et al*, 1998) carcinomas and in other types of cancer (Slamon *et al*, 1987; Scher, 2000), for which overexpression often correlates with a poor prognosis. HER-2/neu is an immunogenic protein because some patients whose tumours overexpress HER-2/neu have pre-existing antibody and T-cell immunity directed against this antigen (Disis and Cheever, 1997). In addition, cytotoxic T lymphocyte (CTL) responses against HER-2/neu^+^ tumours have been induced *in vitro* using major histocompatibility complex (MHC) class I-binding synthetic peptides derived from the HER-2/neu sequence (Fisk *et al*, 1995; Linehan *et al*, 1995; Brossart *et al*, 1998; Rongcun *et al*, 1999; Baxevanis *et al*, 2002). The identification of multiple MHC class I-restricted HER-2/neu-specific CTL epitopes will allow the selection of the epitope with the highest potential for vaccination. Such epitopes must be highly immunogenic and must be able to recruit a wide spectrum of high avidity functional CTL, capable of generating effective antitumour responses.

The HER-2/neu epitope, spanning amino acids 369–377 (HER-2(9_369_)), was first described by Fisk *et al* (1995) as an immunodominant human leucocyte antigen (HLA)-A2-restricted epitope that was recognised by four out of four ovarian tumour-associated lymphocytes, as well as tumour reactive clones. HER-2(9_369_) represents a common epitope expressed by various tumour types including ovarian (Kono *et al*, 1997; Rongcun *et al*, 1999), renal cell (Brossart *et al*, 1998; Seliger *et al*, 2000) and breast (Brossart *et al*, 1998) carcinomas and melanoma cells (Rongcun *et al*, 1999). Tumour cell lines endogenously processing and expressing HER-2(9_369_) in the context of HLA-A2, could be efficiently recognised by the HER-2(9_369_)-specific CTL (Fisk *et al*, 1995; Rongcun *et al*, 1999; Baxevanis *et al*, 2002). In addition, splenocytes from human-CD8 and HLA-A2 transgenic mice that were vaccinated with HER-2(9_369_) recognised human tumour cell lines expressing both HLA-A2 and HER-2/neu (Lustgarten *et al*, 1997). Vaccination of HLA-A2^+^ and HER-2/neu^+^ cancer patients with the helper peptide HER-2(15_369_), which contains the HER-2(9_369_) CTL epitope, resulted in increased frequencies of HER-2(9_369_) precursors and generated CD8^+^ CTL responses against tumour cell lines naturally expressing or pulsed to express HER-2(9_369_) (Knutson *et al*, 2001). In a more recent report, Knutson *et al* (2002) showed that immunisation of four patients with breast or ovarian cancer with HER-2(9_369_) plus GM-CSF resulted in increased peptide-specific T-cell precursor frequencies (PF) in two of them. Active immunisation of patients with HER-2/neu peptide-based vaccines (also including HER-2(15_369_)) generated immunity to HER-2/neu peptides and to HER-2/neu intracellular and extracellular domains (Disis *et al*, 2002). In the same study, patients who received a vaccine consisting of the helper peptides HER-2(15_369_), HER-2(15_688_) and HER-2(15_971_) developed a T-cell response to HER-2(15_369_) but not to the other peptides in the immunising mix, demonstrating the immunodominance of HER-2(15_369_). In our recent work (Baxevanis *et al*, 2002), by applying a CTL induction protocol using patients' dendritic cells (DC) pulsed with total peptide extracts from autologous HER-2/neu^+^ tumours, we could also demonstrate an immunodominance of HER-2(9_369_) in the tumour-specific CTL repertoire of the patients.

Although the immunogenicity of HER-2(9_369_) has been merely shown in studies aiming at generation of peptide-specific CTL *in vitro*, and also *in vivo*, in the course of vaccination protocols assessing the efficacy of the HER-2/neu peptide vaccines, very low PF to this peptide could be detected in HER-2/neu-overexpressing HLA-A2 patients (Disis *et al*, 2000; Knutson *et al*, 2001). Given the fact that HER-2(9_369_) binds with high affinity to HLA-A2 allele, we assumed that T-cell clones recognising this particular bimolecular complex (i.e., HLA-A2+HER-2(9_369_)) might have been partially tolerised, suggesting that boosting or generating an immune response via immunisation could represent a reasonable approach for reactivating such clones. Alternatively, binding of HER-2(9_369_) to alleles other than HLA-A2 allele(s) with intermediate- or low-binding scores would possibly lead to the generation of intermediate- or low-affinity CTL clones not tolerised by the immune apparatus. With regard to the first possibility, Knutson *et al* (2002) demonstrated that HLA-A2 patients immunised with HER-2(15_369_) could develop IFN-*γ* enzyme-linked immunosorbent spot (ELISPOT) responses to HER-2(9_369_) and also exhibited increased HER-2(9_369_)-specific PF. Investigating the second possibility using computer algorithms (SYFPEITHI, www.uni-tuebingen.de/uni/kxi/d atabase.html; Rammensee *et al*, 1999), we found that HER-2(9_369_), in addition to HLA-A2, also binds to HLA-A26 and HLA-A3 alleles with high- (similar to HLA-A2) and intermediate-affinity scores, respectively. Therefore, we thought that it might be important to analyse peptide-specific CTL frequencies in patients with HER-2/neu^+^ tumours expressing these alleles. Besides the binding affinity between peptide and HLA alleles that possibly determine patients' CTL repertoire *in vivo*, an important factor that may influence the determination of PF of *ex vivo* peptide-specific CTL is the short-term *in vitro* stimulation protocol used for detecting peptide-specific cytokine production in ELISPOT assays (Disis *et al*, 2000; Knutson *et al*, 2002). To this end, we used a novel protocol to activate patients' T lymphocytes for evaluating IFN-*γ* in the ELISPOT assay. This included mature DC, generated from patients' monocytes, which were pulsed with HER-2(9_369_) and used, in the presence of IL-7 and IL-12, to stimulate IFN-*γ* production by the autologous peripheral blood mononuclear cells (PBMC). By applying this protocol, we have been able to detect HER-2(9_369_) peptide-specific CTL precursors at high frequencies within PBMC developed mostly in HLA-A3 patients, whereas lower frequencies of the same precursors could be detected in HLA-A2 or HLA-A26 patients.

## PATIENTS AND METHODS

### Patients

Patients with histologically confirmed breast, ovarian, lung, colorectal and prostate carcinomas were included in this study (see also Table 1

**Table 1:** Patients enrolled in this study

**Patient no.**	**Age (years)**	**Sex**	**Type of Ca**	**Stage**	**HLA-A**	**HER-2/neu expression^a^**	**Source of Ca cells**
1	57	F	Breast	III	HLA-A2	1	Solid tumour
2	56	F	Ovarian	III	HLA-A2	2	Solid tumour
3	63	M	Colorectal	III	HLA-A2	1	Solid tumour
4	79	F	Breast	IV	HLA-A2	3	Effusion
5	55	F	Breast	III	HLA-A2	1.5	Solid tumour
6	45	F	Breast	IV	HLA-A2, -A3	2	Effusion
7^b^	47	M	Prostate	IV	HLA-A2	1	Effusion
8	58	F	Breast	III	HLA-A2	2.5	Solid tumour
9	69	F	Ovarian	IV	HLA-A2	2	Solid tumour
10	72	M	Lung	IV	HLA-A2	1	Effusion
11	64	M	Colorectal	III	HLA-A2	2	Effusion
12	55	F	Breast	II	HLA-A2	2	Solid tumour
13	50	F	Colorectal	III	HLA-A2	1	Solid tumour
14	38	F	Lung	IV	HLA-A2	2.5	Effusion
15	45	F	Ovarian	IV	HLA-A2	1.5	Effusion
16	44	F	Ovarian	III	HLA-A2	2	Solid tumour
17	57	F	Breast	III	HLA-A3, -A26	3	Solid tumour
18^b^	62	F	Breast	IV	HLA-A3	2	Effusion
19	75	F	Ovarian	III	HLA-A3	2	Solid tumour
20	67	M	Lung	IV	HLA-A3	1.5	Effusion
21	59	F	Ovarian	IV	HLA-A3	2	Effusion
22	39	F	Lung	IV	HLA-A3, A26	1	Effusion
23	62	M	Prostate	IV	HLA-A3	1	Solid tumour
24	75	F	Breast	III	HLA-A3	3	Solid tumour
25	68	F	Breast	III	HLA-A3	1.5	Solid tumour
26^b^	62	F	Breast	IV	HLA-A26	2	Effusion
27	79	F	Ovarian	III	HLA-A26	1.5	Solid tumour
28	78	F	Ovarian	IV	HLA-A26	2	Effusion
29	65	M	Lung	III	HLA-A26	2.5	Effusion
30	47	M	Colorectal	III	HLA-A26	1.5	Solid tumour
31	49	F	Ovarian	III	HLA-A26	2	Solid tumour
32	57	F	Breast	IV	HLA-A26	3	Effusion
33	57	F	Breast	III	HLA-A2	0	Solid tumour
34	73	F	Breast	IV	HLA-A2, -A3	0	Effusion
35	63	F	Breast	III	HLA-A2	0	Solid tumour
36	72	F	Breast	III	HLA-A2	0	Solid tumour
37	55	M	Lung	IV	HLA-A2	0	Effusion
38	39	F	Ovarian	III	HLA-A2	0	Solid tumour
39	64	M	Prostate	IV	HLA-A2	0	Effusion
40	54	M	Lung	IV	HLA-A2	0	Effusion
41	57	F	Colorectal	III	HLA-A2	0	Solid tumour
42	65	M	Colorectal	III	HLA-A2	0	Solid tumour
43	75	F	Breast	III	HLA-A2	0	Solid tumour
44	63	F	Ovarian	IV	HLA-A2	0	Effusion
45	72	F	Ovarian	III	HLA-A3	0	Solid tumour
46	57	F	Ovarian	IV	HLA-A3	0	Effusion
47	59	F	Breast	III	HLA-A3	0	Solid tumour
48	65	M	Lung	III	HLA-A3	0	Solid tumour
49	47	M	Prostate	III	HLA-A3	0	Solid tumour
50	59	F	Ovarian	III	HLA-A3	0	Solid tumour
51	75	F	Breast	III	HLA-A3	0	Solid tumour
52	69	F	Breast	IV	HLA-A26	0	Effusion
53	75	F	Ovarian	III	HLA-A26	0	Solid tumour
54	73	F	Ovarian	III	HLA-A26	0	Solid tumour
55	59	F	Breast	IV	HLA-A26	0	Solid tumour
56	38	M	Prostate	IV	HLA-A26	0	Effusion
57	39	M	Lung	III	HLA-A26	0	Effusion
58	67	M	Colorectal	III	HLA-A26	0	Solid tumour

a Determined by flow cytometry in effusions and by immunohistochemistry in solid tumours (see also ‘Materials and Methods’).

b Patient homozygous for this allele.

). Peripheral blood mononuclear cells were collected from peripheral blood samples. Biologic material was provided by the Breast Cancer Clinic of Saint Savas Cancer Hospital and the Department of Pathophysiology of Laikon General Hospital under the Institutional Review Board of both institutions. All volunteers provided informed consent before entering these studies.

### Isolation of PBMC

Peripheral blood mononuclear cells were isolated by density gradient centrifugation using Ficoll Separating Solution (Biochrom AG, Berlin, Germany). Cells were washed twice with phosphate-buffered saline (PBS) and used immediately or kept frozen until use.

### HLA-genotyping

Total RNA was extracted from patients' and healthy donors' PBMC using the SV Total RNA Isolation System (Promega, Madison, USA), according to the manufacturer's protocol. First-strand cDNA synthesis was performed using approximately 2 *μ*g of total RNA, oligodT primers and the SuperScript II Rnase H (−) reverse transcriptase (Invitrogen, Carlsbad, USA). This cDNA material was used for PCR amplification with Taq Platinum (Invitrogen) using the PROTRANS Cyclerplate System HLA class I and HLA class II (Protrans, Ketsch, Germany) according to the manufacturer's instructions. The typing results were obtained after electrophoresis of the amplification products on 2% agarose gel containing GelStar dye (FMC BioProducts Rockland, ME, USA) and visualised by UV light. Individuals expressing HLA-A2, HLA-A3 or HLA-A26 alleles or a combination of either were enrolled in this study.

### Peptide synthesis

HER-2 (369–377) (KIFGSLAFL) peptide was synthesised by the solid-phase method with an Ecosyn P peptide synthesiser (Eppendorf-Biotronik, Hamburg, Germany) using the Fmoc strategy and a 4-carboxybenzyl alcohol resin. Purification was performed by high-performance liquid chromatography. The purity was >95%. Quantitative and qualitative determination were controlled by amino-acid analysis and matrix-assisted laser desorption mass spectrophotometry (Kratos Kompact Maldi II, Kratos Analytical, Manchester, UK). Peptide was lyophilised, dissolved in PBS, aliquoted at 2 mg ml^−1^ and stored frozen at −20°C until use.

### Monoclonal antibodies (Abs) and immunophenotyping

The expression of HER-2/neu on tumour cells was determined using the PE-conjugated anti-HER-2/neu monoclonal antibody (mAb) (clone Neu 24.7), which recognises the extracellular domain of HER-2/neu (Becton Dickinson, Mountain View, CA, USA). When malignant effusions were available, the expression of HER-2/neu was determined by flow cytometry comparing the mean fluorescence intensity (MFI) of the tumour cells with the MFI of tumour cell lines expressing HER-2/neu at different levels (HER-2/neu expression of the MDA-231 cell line is scored as 1, of MCF-7 as 2 and of SKBR-3 as 3). The expression of HER-2/neu on solid tumours was performed by immunohistochemistry by estimating the number and intensity of stained tumour cells per section of tumour specimen as previously reported (Berger *et al*, 1989) and using the DAKO's 0–3 scoring system. For DC typing the following mAb were used: anti-CD83 conjugated with PE mAb, obtained from Caltag Laboratories (Burlingame, CA, USA); anti-CD16, -CD20, -CD40 and -CD80 conjugated with FITC and anti-CD3, -CD14, -CD86 and anti-HLA-DR conjugated with PE, purchased from PharMingen (San Diego, CA, USA). Cells to be immunostained were washed twice with ice-cold PBS/1% fetal bovine serum (FBS, Life Technologies, Gaithersburg, MD, USA) followed by incubation with saturating concentrations of the appropriate mAb for 20 min at room temperature. Thereafter, cells were washed twice in ice-cold PBS/1% FBS and fixed with 1% paraformaldehyde in PBS. Samples were analysed using FACSCalibur (Becton Dickinson) and CellQuest analysis software.

### Generation of DC

Dendritic cells were generated from CD14^+^ monocyte precursors purified from freshly isolated PBMC by positive immunoselection using an anti-CD14 mAb coupled onto magnetic microbeads (Miltenyi Biotech, Auburn, CA, USA) under the manufacturer's protocol. Monocyte differentiation in DC was performed as described (Sotiriadou *et al*, 2001). In brief, the CD14^+^ cells were cultured in 2 ml X-VIVO 15 medium (BioWhittaker Europe, Belgium) supplemented with 1% autologous heat-inactivated plasma, 1000 IU ml^−1^ IL-4 (R&D Systems, Europe) and 1000 IU ml^−1^ GM-CSF (Immunex, Seattle, WA, USA). Fresh medium (2 ml) with cytokines was added on days 2 and 4. Tumour necrosis factor-*α* (R&D Systems) was added at 10 ng ml^−1^ on day 6. Dendritic cells were harvested on day 7 and used as antigen-presenting cells (APC) or cryopreserved for later use. The percentage of mature DC recorded was >50%, based on the expression of a CD3^−^, CD14^−^, CD16^−^, CD20^−^, CD40^+^, CD80^+^, CD83^+^, CD86^+^ and HLA-DR^+^ phenotype analysed by flow cytometry (Perez *et al*, 2003). Dendritic cells were used as APC pretreated with 100 *μ*g ml^−1^ mitomycin C (Kyowa, Tokyo, Japan) for 45 min at 37°C. Following an extensive wash in Hank's balanced salt solution (Life Technologies), DC were pulsed with 50 *μ*g ml ^−1^ of the peptide for 4 h at 37°C.

### Enzyme-linked immunosorbent spot assay

The ELISPOT assay was used to determine PF of HER-2(9_369_)-specific CTL. On day 0, PBMC from every individual were plated at 500 000 well^−1^ in quadruplicates in 96 flat-bottom well plates. Autologous DC pulsed with 50 *μ*g ml^−1^ of peptide HER-2(9_369_) were added to PBMC at a cell ratio of 1 : 10 in a total volume of 200 *μ*l well^−1^ X-VIVO 15 medium supplemented with 1% autologous heat-inactivated plasma, 10 ng ml^−1^ human recombinant IL-7 and 100 pg ml^−1^ human recombinant IL-12 (both purchased from R&D systems). Control cultures contained PBMC, stimulated with unpulsed DC or DC pulsed with soluble tetanus toxoid (TT) (Ladecle Laboratories, Pearl River, NY, USA) at 0.1 LFU ml^−1^. Cultures were incubated at 37°C in a CO_2_ incubator. On day 3, 100 *μ*l of the culture supernatant was decanted and replaced by an equal volume of fresh medium supplemented with 20 ng ml^−1^ IL-7 and 200 pg ml^−1^ IL-12. Growing microcultures were restimulated on day 7 with DC pulsed with the same concentration of the peptide or TT. After 24 h, IFN-*γ* production was estimated using the Biosource IFN-*γ* ELISPOT-assay kit (Biosource International, Camarillo, CA, USA) under the manufacturer's protocol. Spots were counted under a stereomicroscope (Zeiss, Germany) using Image ProPlus software (Media Cyhernetics Silver Spring, MD, USA). Specific precursors were calculated by subtracting the mean number of spots obtained from the control cultures (i.e., with unpulsed DC) plus two s.d. from the mean number obtained in the experimental cultures (with peptide-pulsed or TT-pulsed DC). Precursor frequencies were evaluated as the number of specific precursors per 10^6^ PBMC^−1^. Precursor frequencies to HER-2(9_369_) were also enumerated from PBMC from patients with HER-2/neu negative (^−^)tumours and healthy individuals. We considered as responders those HER-2/neu^+^ patients whose individual PF were significantly higher from the mean PF in these two groups.

### Proliferation assay

Proliferative responses to phytohaemagglutinin (PHA) (Sigma) were performed as previously described (Baxevanis *et al*, 1990). Data are presented as stimulation index (i.e., counts per min (c.p.m.) from PBMC cultures with PHA divided by c.p.m. from PBMC cultures without PHA).

### Statistical analysis

Significant differences between individual PF in HER-2/neu^+^ patients with the mean PF in HER-2/neu^−^ patients or healthy donors were assessed by applying *t*-test statistics. Significant differences between the mean PF among groups were also assessed by the *t*-test.

## RESULTS

Patients expressing HLA-A2 (*n*=28), HLA-A3 (*n*=18) or HLA-A26 (*n*=16) were investigated (Table 1). The median time from last chemotherapy was 6 months (range 3–19). Of the breast cancer patients, 13 were diagnosed with stage III disease, 10 with stage IV and one with stage II. Of the ovarian cancer patients, 10 had stage III and six had stage IV disease. All colorectal cancer patients (*n*=7) had stage III disease, whereas of the lung Ca patients, three had stage III and seven of them had stage IV disease. Finally, four patients with prostate cancer were diagnosed as having a stage IV disease and one stage III.

**Figure 1:**
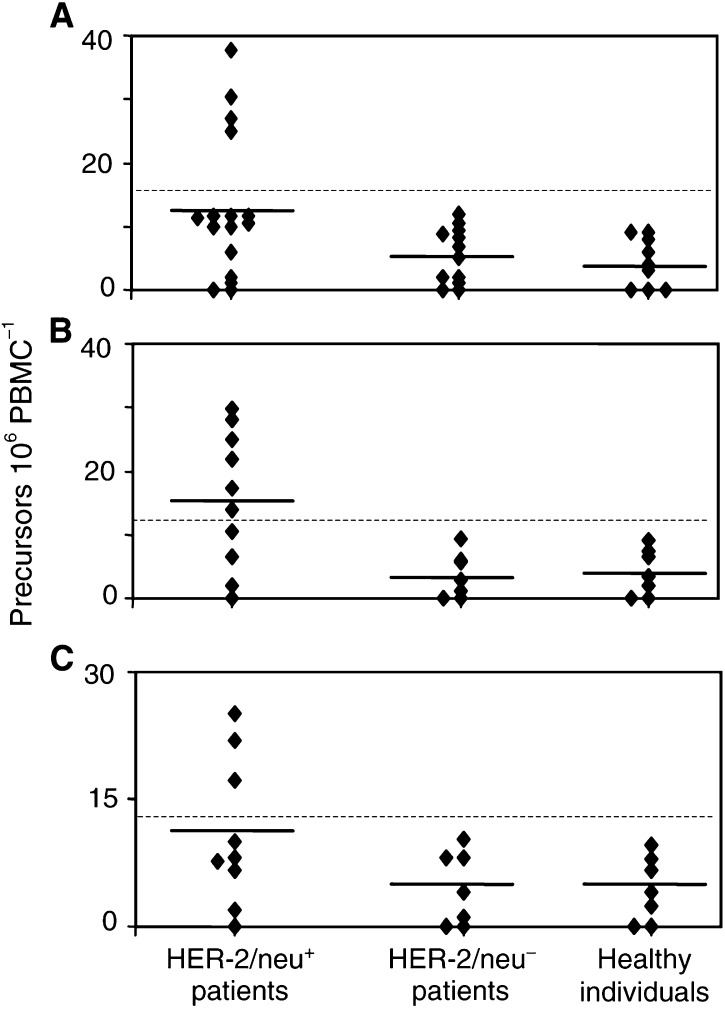
HER-2(9_369_) PF in patients with HER-2/neu^+^ or HER-2/neu^−^ tumours and healthy individuals. Above the dotted line responders to the peptide are shown. The solid lines indicate the mean PF for each group. (**A**) HLA-A2^+^ donors, (**B**) HLA-A3^+^ donors and (**C**) HLA-A26^+^ donors.

Patients enrolled in the study were examined for immunocompetence using IFN-*γ* ELISPOT analysis evaluating the PF to whole TT. Proliferative responses upon stimulation with PHA in the same patients were also estimated. In addition, the HER-2(9_369_)-specific T-cell PF were examined in heterozygous HLA-A2 (*n*=10), HLA-A3 (*n*=7) and HLA-A26 (*n*=7) healthy donors. Four of 16 (25%) HLA-A2 patients (nos. 5, 6, 8 and 12) whose tumours overexpressed HER-2/neu demonstrated CTL immunity to HER-2(9_369_) (range of PF=25.1–37.7; mean PF=30.0, (Figure 1

)) (*P*<0.001 compared to mean PF from HER-2/neu^−^ patients and healthy donors). The rest of HLA-A2, HER-2/neu^+^ patients (*n*=12; 75%) were nonresponders, demonstrating low frequencies of peptide-specific CTL (range of PF=0–11.7; mean PF=7.1, *P*<0.01 compared to mean PF of the four responders), which were almost comparable with those (mean PF) observed in the group of HLA-A2 patients with HER-2/neu^−^ tumours (range of PF=0–11.8; mean PF=5.5) (Figure 1) and of HLA-A2 healthy volunteers (in both cases *P*: nonsignificant (NS)). The frequencies for TT did not differ significantly among patients and healthy donors (range=100.0–476.0; mean: 230.4 for patients and range=108.1–512.8; mean: 170.4 for healthy donors) (*P*=0.25). The magnitude of responses to PHA was also comparable among patients and healthy donors (Data not shown).

The majority of HER-2/neu-overexpressing HLA-A3 patients displayed pre-existing CTL immunity to HER-2(9_369_). As presented in Figure 1, six of 10 patients examined (nos. 6, 17, 18, 19, 22 and 23) demonstrated increased peptide-specific CTL PF (PF range=13.9–29.9; mean PF=22.7). The remaining four patients in this group exhibited low pre-existing CTL PF to HER-2(9_369_) (PF range = 0–10.5; mean PF=4.7, *P*<0.001), which were at almost similar levels with those observed from the groups of HLA-A3, HER-2/neu^−^ patients (range=0–10.5; mean PF=4.5) and of healthy donors (range=0–8.1; mean PF=4.1) (Figure 1). As with HLA-A2 patients, also in this group, responses to TT and PHA were comparable with those of healthy donors (Data not shown).

Finally, we examined HLA-A26 patients with HER-2/neu-overexpressing tumours. Three of nine patients (nos. 17, 22 and 30; 30%) displayed high CTL PF to HER-2(9_369_), ranging from 11.3 to 25.1 (mean PF=21.4), which differed significantly (*P*<0.001) from the PF detected in the residual six nonresponder patients (PF range=0–10.1; mean PF=5.7). These patients along with the seven patients with HER-2/neu^−^ tumours and the seven healthy donors exhibited PF to HER-2(9_369_), which did not differ statistically when compared to each other (PF range and mean PF in HER-2/neu^−^ patients: 0–10.2 and 4.5; and in healthy donors: 0–9.7 and 4.4, respectively). Responses to TT and PHA were similar in all individuals tested (Data not shown).

Finally, by comparing the PF mean values in the group of total HER-2/neu^+^ patients with those observed in HER-2/neu^−^ patients, we found statistically significant differences as follows: HLA-A2^+^ HER-2/neu^+^ *vs* HLA-A2^+^ HER-2/neu^−^, *P*<0.05; HLA-A3^+^ HER-2/neu^+^ *vs* HLA-A3^+^ HER-2/neu^−^, *P*<0.01; HLA-A26^+^ HER-2/neu^+^ *vs* HLA-A26^+^ HER-2/neu^−^, *P*<0.05.

## DISCUSSION

This study provides evidence for the existence of an *in vivo* T-cell response to HER-2(9_369_) in patients with advanced cancer whose tumours overexpress HER-2/neu. Furthermore, our study supports two other points. It demonstrates for the first time that HER-2/neu is a tumour antigen recognised by CTL in prostate cancer patients. Indeed, our HLA-A2, three patients with prostate cancer demonstrated an increased HER-2(9_369_)-specific PF of CTL among PBMC in the IFN-*γ* ELISPOT assay. Second, our results suggest that the HER-2(9_369_) peptide, besides HLA-A2, is also recognised by CTL in the context of two other alleles, namely HLA-A3 and HLA-A26, besides in breast and ovarian cancer patients and also in patients with colorectal, lung and prostate cancer.

A minority of HLA-A2 patients (four of 16; 25%) and of HLA-A26 patients (three of nine; 30%) with HER-2/neu overexpressing cancers in advanced stages had detectable pre-existent CD8^+^ T-cell responses directed against HER-2(9_369_) (in all cases, the frequencies ranged between 31.7 and 17.3). In contrast, the majority of patients with HER-2/neu^+^ tumours carrying the HLA-A3 allele (six of 10; 60%) responded with increased PF to this peptide (range=13.9–29.9). One of the four responders in the HLA-A2 group of patients (Table 1) and two of three responders in the group of patients expressing the HLA-A26 allele (Table 1) also expressed HLA-A3. Taking into consideration the overall responses we obtained, it may be more likely that in these particular cases HLA-A3 would represent the restricting element. Alternatively, these patients may respond to HER-2(9_369_) by virtue of two different sets of T-cell clones restricted by HLA-A2 or HLA-A26 and HLA-A3 alleles.

T cells of HLA-A2 and HLA-A26 patients expressing HER-2/neu had the ability to respond to antigenic stimuli as evidenced by the PHA response, which was comparable with those in the group of HLA-A3 patients, exhibiting high percentages of donors with peptide-specific pre-existent immunity, and in the group of healthy donors. Furthermore, this study group had a similar incidence of immune responses to TT as described in the population of healthy donors. Thus, one reason that may account for the low numbers of peptide-specific responders in the groups of HLA-A2 and HLA-A26 patients is that HER-2(9_369_) has a high binding score to the HLA-A2 and HLA-A26 alleles, whereas it binds only with intermediate affinity to HLA-A3 (Database SYFPEITHI; Rammensee *et al*, 1999). Consequently, during natural immunogenic processing of the intact HER-2/neu protein, HER-2(9_369_) may be presented in the context of HLA-A2 or HLA-A26 in an immunodominant manner and be recognised by high-affinity CD8^+^ T-cell clones, which will be tolerised by the immune system in order to prevent the induction of an immune response to nonmalignant epithelial cells also expressing HER-2/neu. Indeed, dominantly processed self-determinants are thought to be efficient in tolerance induction (Nanda and Secarz, 1995; Sercarz, 2000).

Conversely, the high percentages of HER-2(9_369_)-specific precursors that we observed in HLA-A3 patients may be explained by the fact that, due to its capacity to bind with intermediate affinity to this particular allele, HER-2(9_369_) may be recognised by low-affinity CD8^+^ T-cell clones. Such clones are not tolerised, because they will not recognise basal levels of HER-2/neu expressed in the normal epithelium. However, the abundance of this peptide-epitope in HLA-A3 molecules expressed either on syngeneic tumour cells or HER-2(9_369_)-pulsed syngeneic DC will activate these clones to produce cytokines (e.g. IFN-*γ*) and lyse their specific targets.

The state of tolerance to self-antigens may be circumvented *in vivo* by peptide-based vaccinations or *in vitro* by repetitive restimulations with peptide-pulsed autologous DC, as has been already shown for melanoma differentiation antigens (Jager *et al*, 2002) and also HER-2/neu. With respect to the latter, most of HLA-A2 breast cancer patients with HER-2/neu-overexpressing tumours receiving monthly injections with the helper peptide HER-2(15_369_), which contains within the putative HLA-A2-binding motif HER-2(9_369_), developed, after immunisation, CD8 T-cell responses to peptide HER-2(9_369_) (10 of 15 patients tested; 66.6%) (Knutson *et al*, 2001). Before immunisation only two of 15 (i.e., 13.33%) expressed pre-existent immunity to this peptide (Knutson *et al*, 2001). A similar situation was reported by Disis *et al* (2002), where the vast minority of HLA-A2 patients had pre-existent immune responses to HER-2(9_369_) (two of 38; 5%), whereas the majority of these patients developed peptide-specific immunity upon stimulation with HER-2(15_369_). More strikingly, breast and ovarian cancer patients immunised with HER-2(9_369_) developed T-cell precursors specific for this peptide (Knutson *et al*, 2002). Since in these immunisation protocols patients enrolled expressed HLA-A2, it remains to be examined whether a similar situation will also appear with HLA-A26 and HLA-A3 patients.

Our results demonstrate higher percentages (25%) of HLA-A2 patients with pre-existing immunity to HER-2/neu peptide compared to those (i.e., 13.33, 5 and 0%) reported by others (Disis *et al*, 2000,^2002^; Knutson *et al*, 2001). Since an IFN-*γ* ELISPOT assay was used to determine PF of peptide-specific CD8^+^ T lymphocytes, we believe that the differences in our and their protocols may account for such discrepancies. First of all, we must point out the fact that we, as they also did, estimated PF from PBMC and not isolated CD8^+^ T cells. However, since it is well established that HER-2(9_369_) is recognised by MHC class I-restricted CD8^+^ CTL (as it is the case with peptides consisting of 8–10 aminoacids) (Fisk *et al*, 1995; Brossart *et al*, 1998; Rongcun *et al*, 1999; Baxevanis *et al*, 2002), we can be sure that we measured CD8^+^ T-cell PF. Regarding culture conditions during the incubation period, it is essential to note that we used autologous DC (instead of PBMC) as peptide-presenting cells in the presence of exogenously added IL-7 and IL-12 (instead of IL-2) both of which are known to support antigen-specific CD8^+^ T-cell responses (Costello *et al*, 1993; Trinchieri, 1995,^1997^). In this way, we may have established a culture system favouring the detection of pre-existing CD8^+^ T cell-mediated responses, eventually also in individuals who have developed physiological mechanisms of immunologic tolerance.

To this end, it is important to mention that preliminary experiments performed in our laboratory demonstrated the capacity of cultures with high peptide-specific CTL PF to lyse autologous peptide-pulsed, but not unpulsed DC as well as autologous tumour cells.

There are numerous examples of CTL that are stimulated with synthetic peptides (derived from the sequence of tumour antigens) which are able to recognise and kill tumour cells effectively (for a review, see Wang and Rosenberg, 1999). In prostate cancer, several markers such as prostate-specific antigen, prostatic acid phosphatase, prostate stem cell antigen and prostate-specific membrane antigen, which are all preferentially expressed by prostatic epithelial cells, have been demonstrated to serve as substrate sources of immunogenic peptide epitopes recognised by CTL (Correale *et al*, 1998; Peshwa *et al*, 1998; Dannull *et al*, 2000; Lu and Celis, 2002). HER-2/neu has also been identified to be expressed on prostate cancer cells (Scher, 2000). Herein, we show that one patient with prostate cancer developed increased CTL PF for HER-2(9_369_), suggesting that this epitope is naturally processed and expressed on prostate tumour cells. McNeel *et al* (2003) vaccinated patients with advanced prostate cancer with the E75 HLA-A2 epitope from HER-2/neu (i.e., HER-2(9_369_)) using flt3 ligand as an adjuvant. Apart from a single patient (20 patients were enrolled in their study), no significant peptide-specific T-cell responses could be detected by ELISPOT. These data, although demonstrating that only a minority of HLA-A2^+^ prostate cancer patients responded to this peptide, still do not exclude the possibility that by using alternative immunisation protocols (e.g., injections with peptide-pulsed DC plus flt3 ligand) the number of responders might be considerably increased.

In summary, we have evaluated HER-2(9_369_)-specific PF in patients with five different types of cancer. Patients with pre-existing immunity to this peptide have been scored, in addition to breast and ovarian, also in colorectal, lung and, for the first time, in prostate cancer, demonstrating that HER-2(9_369_) is ideal for peptide-based vaccinations in these types of cancer. Our unpublished observations that T cells from HLA-A2, A3 and A26 patients with pre-existing immunity to HER-2(9_369_) could also lyse their HER-2/neu^+^ autologous tumours points to the fact that this peptide is endogenously processed and presented in the surface of tumours by any of the three alleles, thus increasing the population of patients that can be enrolled in peptide-based vaccinations.

## References

[bib1] Bargmann CI, Hung MC, Weinberg RA (1986) The neu oncogene encodes an epidermal growth factor receptor-related protein. Nature (Lond) 319: 226–230 3945311 10.1038/319226a0

[bib3] Baxevanis CN, Gritzapis AD, Tsitsilonis OE, Katsoulas HL, Papamichail M (2002) HER-2/neu-derived peptide epitopes are also recognized by cytotoxic CD3(+)CD56(+) (natural killer T) lymphocytes. Int J Cancer 98: 864–872 11948464 10.1002/ijc.10251

[bib4] Baxevanis CN, Reclos GJ, Papamichail M (1990) Decreased HLA-DR antigen expression on monocytes causes impaired suppressor cell activity in multiple sclerosis. J Immunol 144: 4166–4171 2140389

[bib6] Berger U, Wilson P, Thethi S, McClelland RA, Greene GL, Coombes RC (1989) Comparison of an immunocytochemical assay for progesterone receptor with a biochemical method of measurement and immunocytochemical examination of the relationship between progesterone and estrogen receptors. Cancer Res 49: 5176–5179 2766287

[bib7] Brossart P, Stuhler G, Flad T, Stevanovic S, Rammensee HG, Kanz L, Brugger W (1998) Her-2/neu-derived peptides are tumor-associated antigens expressed by human renal cell and colon carcinoma lines and are recognized by *in vitro* induced specific cytotoxic T lymphocytes. Cancer Res 58: 732–736 9485028

[bib8] Correale P, Walmsley K, Zaremba S, Zhu M, Schlom J, Tsang KY (1998) Generation of human cytolytic T lymphocyte lines against prostate-specific antigen (PSA) employing a PSA oligoepitope peptide. J Immunol 161: 3186–3194 9743387

[bib9] Costello R, Imbert J, Olive D (1993) Interleukin-7, a major T-lymphocyte cytokine. Eur Cytokine Network 4: 253–262 8268415

[bib10] Coussens L, Yang-Feng TL, Liao YC, Chen E, Gray A, McGrath J, Seeburg PH, Libermann TA, Schlessinger J, Francke U, Levinson A, Ullrich A (1985) Tyrosine kinase receptor with extensive homology to EGF receptor shares chromosomal location with neu oncogene. Science (Wash DC) 230: 1132–1139 10.1126/science.2999974 2999974

[bib11] Dannull J, Diener PA, Prikler L, Furstenberger G, Cerny T, Schmid U, Ackermann DK, Groettrup M (2000) Prostate stem cell antigen is a promising candidate for immunotherapy of advanced prostate cancer. Cancer Res 60: 5522–5528 11034097

[bib12] Disis ML, Cheever MA (1997) HER-2/neu protein: a target for antigen-specific immunotherapy of human cancer. Adv Cancer Res 71: 343–371 9111870 10.1016/s0065-230x(08)60103-7

[bib13] Disis ML, Gooley TA, Rinn K, Davis D, Piepkorn M, Cheever MA, Knutson KL, Schiffman K (2002) Generation of T-cell immunity to the HER-2/neu protein after active immunization with HER-2/neu peptide-based vaccines. J Clin Oncol 20: 2624–2632 12039923 10.1200/JCO.2002.06.171

[bib14] Disis ML, Knutson KL, Schiffman K, Rinn K, McNeel DG (2000) Pre-existent immunity to the HER-2/neu oncogenic protein in patients with HER-2/neu overexpressing breast and ovarian cancer. Breast Cancer Res Treat 62: 245–252 11072789 10.1023/a:1006438507898

[bib15] Fisk B, Blevins TL, Wharton JT, Ioannides CG (1995) Identification of an immunodominant peptide of HER-2/neu protooncogene recognized by ovarian tumor-specific cytotoxic T lymphocyte lines. J Exp Med 181: 2109–2117 7539040 10.1084/jem.181.6.2109 PMC2192068

[bib16] Jager E, Jager D, Knuth A (2002) Clinical cancer vaccine trials. Curr Opin Immunol 14: 178–182 11869889 10.1016/s0952-7915(02)00318-7

[bib17] Knutson KL, Schiffman K, Cheever MA, Disis ML (2002) Immunization of cancer patients with a HER-2/neu, HLA-A2 peptide, p369–377, results in short-lived peptide-specific immunity. Clin Cancer Res 8: 1014–1018 12006513

[bib18] Knutson KL, Schiffman K, Disis ML (2001) Immunization with a HER-2/neu helper peptide vaccine generates HER-2/neu CD8 T-cell immunity in cancer patients. J Clin Invest 107: 477–484 11181647 10.1172/JCI11752 PMC199268

[bib19] Kono K, Halapi E, Hising C, Petersson M, Gerdin E, Vanky F, Kiessling R (1997) Mechanisms of escape from CD8+ T-cell clones specific for the HER-2/neu proto-oncogene expressed in ovarian carcinomas: related and unrelated to decreased MHC class I expression. Int J Cancer 70: 112–119 8985099 10.1002/(sici)1097-0215(19970106)70:1<112::aid-ijc17>3.0.co;2-n

[bib20] Linehan DC, Goedegebuure PS, Peoples GE, Rogers SO, Eberlein TJ (1995) Tumor-specific and HLA-A2-restricted cytolysis by tumor-associated lymphocytes in human metastatic breast cancer. J Immunol 155: 4486–4491 7594611

[bib21] Lu J, Celis E (2002) Recognition of prostate tumor cells by cytotoxic T lymphocytes specific for prostate-specific membrane antigen. Cancer Res 62: 5807–5812 12384542

[bib22] Lustgarten J, Theobald M, Labadie C, LaFace D, Peterson P, Disis ML, Cheever MA, Sherman LA (1997) Identification of Her-2/neu CTL epitopes using double transgenic mice expressing HLA-A2.1 and human CD.8. Hum Immunol 52: 109–118 9077559 10.1016/S0198-8859(96)00292-3

[bib23] Maxwell-Armstrong CA, Durrant LG, Scholefield JH (1998) Colorectal cancer vaccines. Br J Surg 85: 149–154 9501807 10.1046/j.1365-2168.1998.00704.x

[bib24] McNeel DG, Knutson KL, Schiffman K, Davis DR, Caron D, Disis ML (2003) Pilot study of an HLA-A2 peptide vaccine using flt3 ligand as a systemic vaccine adjuvant. J Clin Immunol 23: 62–72 12645861 10.1023/a:1021904432489

[bib25] Nanda NK, Secarz EE (1995) Induction of anti-self-immunity to cure cancer. Cell 82: 13–17 7606778 10.1016/0092-8674(95)90047-0

[bib26] Perez SA, Sotiropoulou PA, Gkika DG, Mahaira LG, Niarchos DK, Gritzapis AD, Kavalakis YG, Antsaklis AI, Baxevanis CN, Papamichail M (2003) A novel myeloid-like NK cell progenitor in human umbilical cord blood. Blood 101: 3444–3450 12506032 10.1182/blood-2002-05-1501

[bib27] Peshwa MV, Shi JD, Ruegg C, Laus R, van Schooten WC (1998) Induction of prostate tumor-specific CD8^+^ cytotoxic T-lymphocytes *in vitro* using antigen-presenting cells pulsed with prostatic acid phosphatase peptide. Prostate 36: 129–138 9655265 10.1002/(sici)1097-0045(19980701)36:2<129::aid-pros8>3.0.co;2-d

[bib28] Rammensee H, Bachmann J, Emmerich NP, Bachor OA, Stevanovic S (1999) SYFPEITHI: database for MHC ligands and peptide motifs. Immunogenetics 50: 213–219 10602881 10.1007/s002510050595

[bib29] Rongcun Y, Salazar-Onfray F, Charo J, Malmberg KJ, Evrin K, Maes H, Kono K, Hising C, Petersson M, Larsson O, Lan L, Appella E, Sette A, Celis E, Kiessling R (1999) Identification of new HER2/neu-derived peptide epitopes that can elicit specific CTL against autologous and allogeneic carcinomas and melanomas. J Immunol 163: 1037–1044 10395702

[bib30] Scher HI (2000) HER2 in prostate cancer–a viable target or innocent bystander? J Natl Cancer Inst 92: 1866–1868 11106669 10.1093/jnci/92.23.1866

[bib31] Seliger B, Rongcun Y, Atkins D, Hammers S, Huber C, Storkel S, Kiessling R (2000) HER-2/neu is expressed in human renal cell carcinoma at heterogeneous levels independently of tumor grading and staging and can be recognized by HLA-A2.1-restricted cytotoxic T lymphocytes. Int J Cancer 87: 349–359 10897039

[bib32] Sercarz EE (2000) Driver clones and determinant spreading. Autoimmun 14: 275–277 10.1006/jaut.2000.0380 10882052

[bib33] Slamon DJ, Clark GM, Wong SG, Levin WJ, Ullrich A, McGuire WL (1987) Human breast cancer: correlation of relapse and survival with amplification of the HER-2/neu oncogene. Science (Wash DC) 235: 177–182 10.1126/science.3798106 3798106

[bib34] Slamon DJ, Leyland-Jones B, Shak S, Fuchs H, Paton V, Bajamonde A, Fleming T, Eiermann W, Wolter J, Pegram M, Baselga J, Norton L (2001) Use of chemotherapy plus a monoclonal antibody against HER2 for metastatic breast cancer that overexpresses HER2. N Engl J Med 344: 783–792 11248153 10.1056/NEJM200103153441101

[bib35] Sotiriadou R, Perez SA, Gritzapis AD, Sotiropoulou PA, Echner H, Heinzel S, Mamalaki A, Pawelec G, Voelter W, Baxevanis CN, Papamichail M (2001) Peptide HER2 (776–788) represents a naturally processed broad MHC class II-restricted T cell epitope. Br J Cancer 85: 1527–1534 11720440 10.1054/bjoc.2001.2089 PMC2363935

[bib36] Trinchieri G (1995) Interleukin-12: a proinflammatory cytokine with immunoregulatory functions that bridge innate resistance and antigen-specific adoptive immunity. Annu Rev Immunol 13: 251–278 7612223 10.1146/annurev.iy.13.040195.001343

[bib37] Trinchieri G (1997) Cytokines acting on or secreted by macrophages during intracellular infection. Curr Opin Immunol 9: 17–21 9039773 10.1016/s0952-7915(97)80154-9

[bib38] Wang RF, Rosenberg SA (1999) Human tumor antigens for cancer vaccine development. Immunol Rev 170: 85–102 10566144 10.1111/j.1600-065x.1999.tb01331.x

[bib39] Yamanaka Y, Friess H, Kobrin MS, Buchler M, Kunz J, Beger HG, Korc M (1993) Overexpression of HER2/neu oncogene in human pancreatic carcinoma. Hum Pathol 24: 1127–1134 8104858 10.1016/0046-8177(93)90194-l

